# Survival Analysis in Cutaneous Leishmaniasis Patients: A Retrospective Cohort Study

**DOI:** 10.3390/biomedicines14081762

**Published:** 2026-08-05

**Authors:** José Fabrício de Carvalho Leal, Caio César Bezerra Silva, Maria Beatriz Souza Nascimento, Renata Trindade Gonçalves, Lucia Helena Gomes Fernandes, Aridne Souza Costa Campos, Fellipe Magela de Araújo, Raimunda Nonata Ribeiro Sampaio, Daniel Holanda Barroso

**Affiliations:** 1 Postgraduate Program in Tropical Medicine, University of Brasília (UnB), Brasília 70910-900, Brazil; fabriciolealc29@gmail.com; 2 Faculty of Medicine, University of Brasília (UnB), Brasília 70910-900, Brazil; contato.caiocesarbezerrasilva@gmail.com (C.C.B.S.); beatriz.maria.unb@gmail.com (M.B.S.N.); 3 Postgraduate Program in Medical Sciences, University of Brasília (UnB), Brasília 70910-900, Brazil; tatinhatg@gmail.com (R.T.G.); luciahelenagfster@gmail.com (L.H.G.F.); aridnecampos@gmail.com (A.S.C.C.); fellipemagela@gmail.com (F.M.d.A.); rnrsampaio@hotmail.com (R.N.R.S.); 4 University Hospital of Brasília (HUB), University of Brasília (UnB), Brasília 70910-900, Brazil; 5 Postgraduate Program in Health Sciences, University of Brasília (UnB), Brasília 70910-900, Brazil

**Keywords:** disseminated leishmaniasis, survival analysis, treatment, *Leishmania* (*Viannia*) *braziliensis*

## Abstract

**Background/Objectives**: American Tegumentary leishmaniasis typically presents as a single ulcerative lesion, but atypical clinical forms exist with diverse physiopathology and treatment responses. Disseminated cutaneous leishmaniasis is a less common presentation that is often challenging to treat and for which evidence-based clinical decisions are scant. A better understanding of treatment response may support the development of alternative therapeutic strategies. This study reports a retrospective cohort analysis using a time-to-event outcome to compare disseminated leishmaniasis with localized cutaneous leishmaniasis using a multivariate model. **Methods**: A total of 366 patients were screened for inclusion between 2014 and 2024, of whom 123 met the inclusion criteria. **Results**: After adjustment for age and sex, patients with disseminated cutaneous leishmaniasis had a significantly longer time to healing than those with localized cutaneous leishmaniasis (HR = 0.29; *p* = 0.021). **Conclusions**: Disseminated cutaneous leishmaniasis seems to be an important predictor of poorer clinical outcomes with prolonged healing time. New therapeutic strategies are urgently needed to address this issue.

## 1. Introduction

American tegumentary leishmaniasis (ATL) is an infectious, non-contagious, neglected and emerging tropical disease caused by protozoa of the genus *Leishmania*, transmitted by the bite of infected female sandflies. ATL is an important public health problem and can present as localized (CL) or disseminated (DL) disease, as well as more severe forms, such as mucocutaneous (ML) and diffuse cutaneous (DCL). This broad clinical spectrum is influenced by parasite species, the host’s immune response, and environmental factors [1,2,3].

DL is mainly caused by *Leishmania* (*Viannia*) *braziliensis*, characterized by the presence of numerous (tens to hundreds or thousands) of polymorphic skin lesions, in contrast to the single or few well-defined ulcerated lesions with raised borders presented in CL. Patients frequently report the initial appearance of a single lesion, usually in the extremities, with subsequent cutaneous dissemination that may affect extensive areas of the body, occasionally accompanied by fever and chills. The pathogenesis of DL involves a complex interplay between parasite and host factors, in addition to being considered more difficult to treat and characterized by frequent relapses [4,5,6,7,8].

Treatment of ATL often requires prolonged regimens and, in many cases, multiple lines of therapy. In CL, management often involves pentavalent antimonials (N-methyl-glucamine antimoniate [Glucantime^®^] and sodium stibogluconate [Pentostam^®^]) administered systemically or intralesionally, pentamidine isethionate, paromomycin, liposomal or deoxycholate amphotericin B, thermotherapy, and, in selected cases, miltefosine. Cure rates in CL are generally higher than those observed in the extensive forms. DL, on the other hand, requires systemic treatment, usually associating pentavalent antimonials, liposomal amphotericin B or miltefosine, seeking to increase treatment efficacy and reduce toxicity; in addition, hospital-based monitoring is indicated due to potential adverse events and the need for dose adjustments [1,7,8,9].

The therapeutic challenge in DL is due to the large number of skin lesions, the dysregulated host immune response, and possibly differences in the parasite species. Consequently, healing is delayed, with higher rates of primary failure, recurrence, and need for retreatment than in CL. Studies indicate longer re-epithelialization times and a significantly higher cumulative probability of failure, especially when there is a delay in the start of treatment or comorbidities [7,10,11].

In this context, most studies evaluating the treatment of DL are observational, without a comparator group, involve small sample sizes, lack appropriate comparator groups, and use heterogeneous therapeutic regimens (e.g., type of drug and treatment duration) and outcome definitions. Time-to-event methods, such as Kaplan–Meier survival analysis and Cox proportional hazards models, are especially well suited to this setting because they account for both the occurrence and timing of cure while accommodating differences in follow-up time and censoring. These approaches are especially valuable for rare conditions such as DL, in which maximizing the information obtained from relatively small sample sizes is essential. Therefore, we conducted a retrospective cohort study using survival analysis to compare the time to healing of the main therapeutic regimens and to compare outcomes between CL and DL.

## 2. Materials and Methods

### 2.1. Population and Case Definition

In this retrospective cohort study, we screened new patients who attended between 2014 and 2024 at the leishmaniasis clinic of the University Hospital of Brasilia (Midwest region of Brazil). Standardized clinical evaluation was performed according to the clinic’s routine protocol. We included patients with cutaneous lesions diagnosed as leishmaniasis according to the composite reference standard that involved the detection of *Leishmania* sp. in at least one of the following exams—direct microscopic examination, culture, polymerase chain reaction (PCR) or histopathological examination; or at least two of the following supportive tests: serology, the Leishmania skin test, or histopathological examination. Complementary exam methodology is described elsewhere [12]. Patients with an alternative diagnosis, those without cutaneous lesions and those who were lost to follow-up before treatment initiation were excluded.

### 2.2. Ethics

This study has been approved by the University of Brasilia ethics committee under the number CAAE 65790722.1.0000.5558.

### 2.3. Groups, Outcomes and Covariates

The primary outcome in the time-to-event analysis was cure. Cure was defined as complete re-epithelialization of all lesions, with complete flattening and absence of erythema. Subjects were split into two groups: DL, which was characterized as the presence of 10 or more cutaneous lesions in at least two body segments as defined elsewhere [13], and localized cutaneous leishmaniasis (CL). According to the institutional protocol, patients were evaluated weekly during treatment and at least monthly after treatment until cure was achieved. Collected covariates included age, sex, time between lesion onset and beginning of treatment and treatment regimen (meglumine antimoniate, amphotericin B, miltefosine and/or pentamidine). Data were extracted from the electronic medical record system following the Leishmaniasis clinic institutional protocol.

### 2.4. Statistical Analysis

The primary outcome, cure, was assessed in a time-to-event analysis. According to the intention-to-treat principle, data on all participants included were analyzed until the outcome or censoring. Differences between DL and CL groups were evaluated using a Cox proportional hazards model and hazard ratios (HRs), and Wald *p* values are reported. A second multivariable model with adjustment for the studied covariates was also built. The distribution of categorical variables was compared between groups using the Fisher exact test. Continuous variables were compared using Student’s *t*-test in case of normal distribution and Wilcoxon rank-sum otherwise. Post hoc power analysis was performed in this convenience sample study, arriving at 0.41 power to detect a 0.05 two-sided type I error rate with a 0.35 hazard ratio, considering the number of subjects effectively included in this study. All analyses were performed in RStudio: Integrated Development Environment for R, Version 2025.05.1 + 513 (RStudio Team, Boston, MA, USA).

## 3. Results

**Figure 1:**
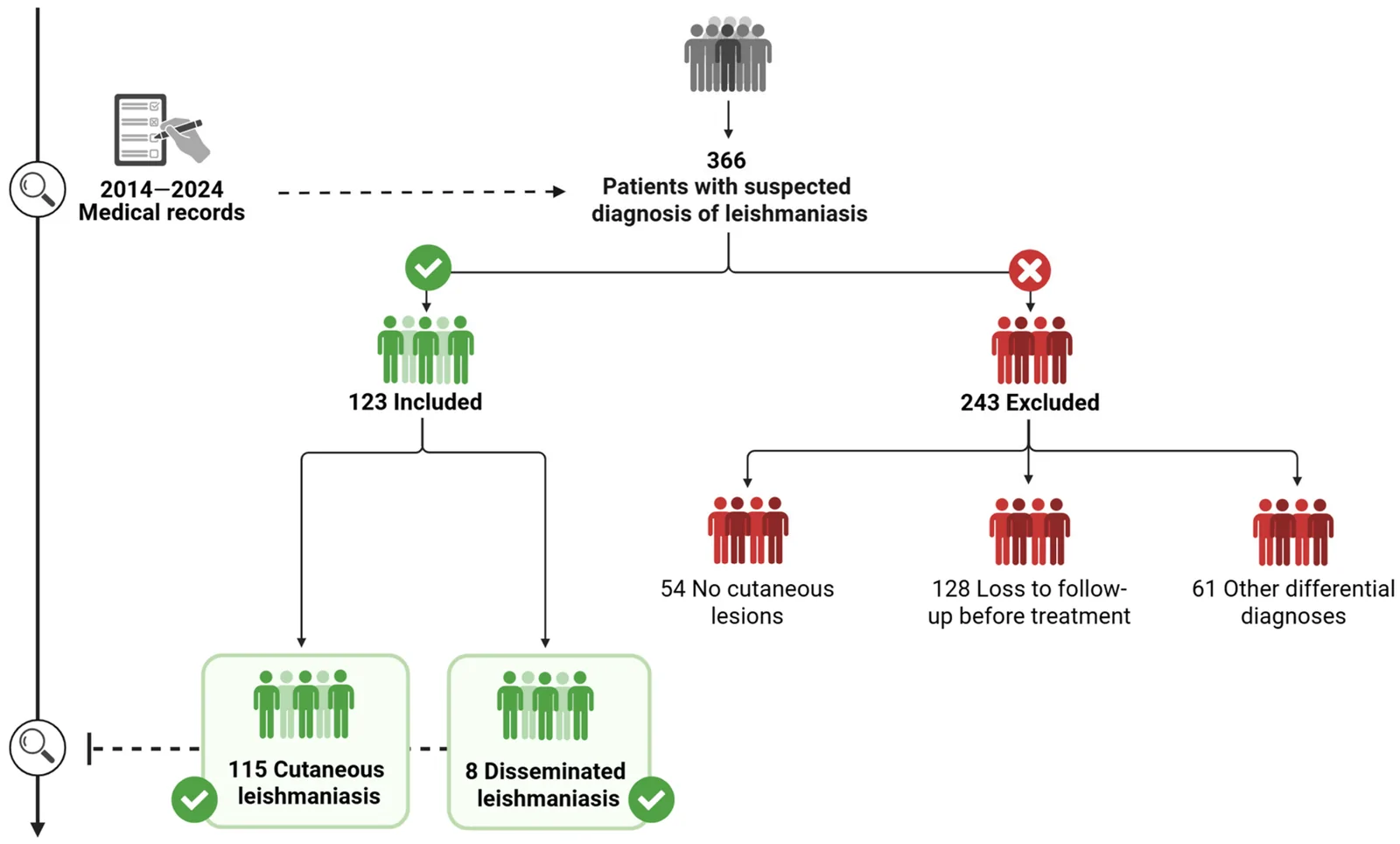
Flow chart of patient eligibility assessment and allocation.

From the 366 patients evaluated at the Leishmaniasis Clinic between 2014 and 2024, a total of 123 met the criteria and were included in this study. From these, 115 (93.4%) had CL and 8 (6.5%) had DL (Figure 1). Of the 243 who did not meet the criteria, 54 had isolated mucosal lesions, 61 had an alternative diagnosis, and 128 were lost to follow-up before the initiation of treatment (Figure 1).

### 3.1. Patient Characteristics

Patient characteristics at baseline are presented in Table 1. Mean age was 44.6 years (IC 95% = 41.4–47.8) in the CL group and 45.0 years (IC 95% = 30.9–59.0) in the DL group. Median lesion duration was 4 months (IQR = 3–6) being 4 months CL group and 5 months (IQR = 3–6.75) in the DL group. Patients with disseminated leishmaniasis had significantly higher chances of being treated with amphotericin B, 50% versus 10% in the CL group, and with pentamidine, 25% versus 3.5% in those with CL. All patients with disseminated leishmaniasis were male, versus 90% in the CL group. Meglumine antimoniate (Glucantime^®^) was the most frequently used treatment in both groups, being used in statistically similar proportions with CL (87.5%) and DL (87.8%). The proportion of patients who used miltefosine was also not significantly different between groups (Table 1).

### 3.2. Outcome

**Figure 2:**
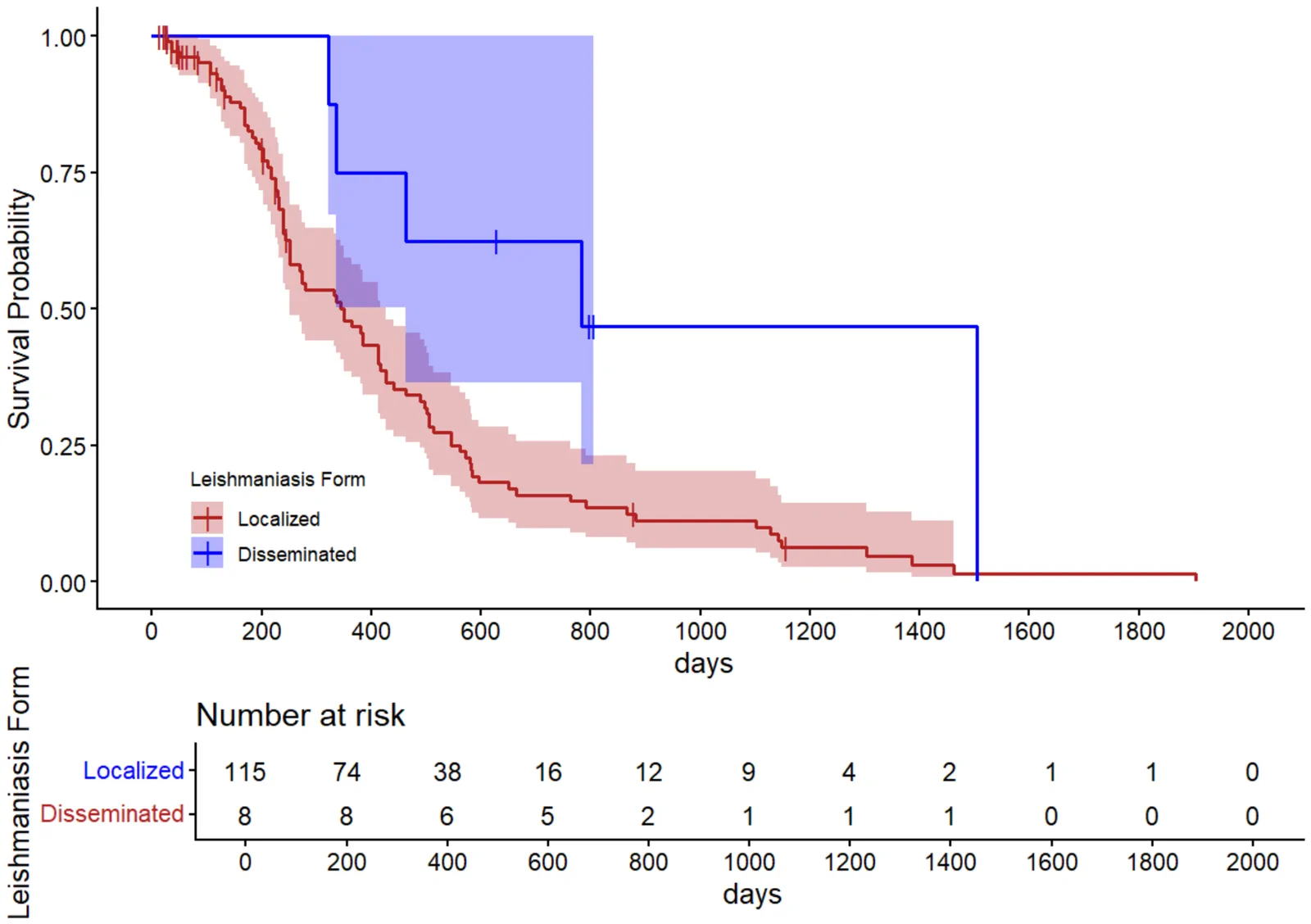
Survival curve of patients with tegumentary leishmaniasis according to disease form (CL versus DL). Shaded areas correspond to CI 95%.

In the survival analysis using a univariate Cox proportional hazards model, patients with DL had a significantly prolonged time to cure, HR = 0.35, *p* = 0.024 (Figure 2). This association between disease form and healing time persisted after adjusting for age, sex and disease duration (HR = 0.29; *p* = 0.021). The use of amphotericin B was also associated with significantly longer healing time than other treatment regimens. Median healing time was 364 days, being 350 days in patients with CL and 784 days in patients with DL.

## 4. Discussion

Here we opted to examine the influence of clinical form on treatment outcomes in an *L.* (*V.*) *braziliensis* area. Due to its unusual physiopathology and response to treatment [14,15], DL is usually excluded from clinical trials [16,17]. Thus, studies on this disease form, such as ours, are an important step to guide clinical practice in an evidence-based manner.

A proportion of 6.5% of all patients in our cohort had DL. Worldwide, this form represents less than 1% of cutaneous leishmaniasis cases [18]. In Brazil, this proportion seems to be increasing, representing 0.2% in earlier case series [19] and stepping up to 3.9 to 5.4% in more recent years [5,20]. The greater proportion of this clinical form in this study may reflect this increase in prevalence, but it can also be related to the fact that it was conducted in a tertiary hospital and patients with more severe and unusual clinical forms are more likely to be referred to our center. Additionally, patients with DL could have a lower propensity to be lost to follow-up before the diagnosis was confirmed as a result of their higher perceived severity of disease and lower chance of spontaneous cure. This could in turn result in attrition bias [21] with a higher prevalence of this form in the population studied.

In this study, we have observed a median healing time of 364 days, which is shown to be longer than that observed in other studies, which varied from 77 to 176 days [22,23]. This discrepancy may be explained by the heterogeneity between studies associated with the characteristics of the population relating both to their basic characteristics, but also to the offered treatment and the most prevalent *Leishmania* species in the region. In areas where *Leishmania major* is the dominant species, CL cured spontaneously in a median of 77.7 days [23]. In a *L.* (*V.*) *braziliensis* endemic area, however, complete healing took a median time of 114 days, ranging from 7 to 474 days [24], being similar to the median 102 days that was observed in a *L.* (*L.*) *amazonensis* area [25]. Importantly, treatment seems to be fundamental to achieve shorter healing times, especially in *L.* (*V.*) *brasiliensis* CL, as only 6.4% of cases heal spontaneously according to a meta-analysis on the subject [26]. Cure criteria may also be an important factor in explaining our results. In clinical trials, it has been suggested that the complete reepithelialization of ulcerated lesions or complete flattening in non-ulcerated lesions should be used to define clinical cure at 90 days [27]. In our clinical practice, however, it is not uncommon to offer a new treatment to patients who, despite complete reepithelialization, have significant raising of lesion borders. Consequently, we opted to consider cured only patients who had complete reepithelialization and complete flattening of the lesion. As cutaneous leishmaniasis tends to re-epithelialize before it is completely flat [24], cure criteria can be one of the main factors to explain the longer healing time we observed in our study.

DL disease form had a significantly higher time to cure HR = 0.35 when compared to CL. Previous studies had already suggested that this form has a worse prognosis. Studies done in *L.* (*V.*) *braziliensis* areas have shown that three-fourths of DL patients fail standard treatment regimens [5]. Cure rates with a single treatment seem to be different in the *Leishmania* (*Viannia*) *panamensis* area, reaching 86.3% in a study from Colombia [28]. However, comparative studies between DL and CL are scarce. In a large case–control study from Brazil’s northwest, Machado et al. have shown a lower cure rate and higher cure time for patients with DL without matching for age and sex [7]. The scarcity of clinical studies on this form is further aggravated by the limited generalizability of results of studies done in different areas related to prevalent species.

Despite not reaching statistical significance, we observed a higher proportion of male patients in the DL group. This higher proportion of male patients within the DL subgroup has already been shown in numerous studies with diverse designs. In a case series comparing *L.* (*V.*) *braziliensis* isolates, 87.5% of subjects with DL were male, compared with only 70.8% of those with CL [14]. In a large retrospective study from northwestern Brazil, the ratio between male and female patients with DL was 8.7:1 compared to 1.7:1 in CL patients [20]. The high proportion of male to female ratio is also evident in case series from other locations such as Colombia 5.75:1 [28] and Brazil’s Southwest 18:1 [5]. It is, thus, not surprising that no female patient fulfilled the criteria to be included in the DL. Considering this association between DL and male sex, we opted to control for male sex in the multivariate analysis.

Although sometimes showing similar effect sizes, compared to case–control studies, cohort studies are less susceptible to selection and recall bias [29]. Additionally, by controlling covariates, we were able to better estimate the effect of the clinical form on prognosis. Having said that, we were not able to control the use of amphotericin in the analysis since there was a significantly higher proportion of patients in the DL group who used amphotericin. This can be a potential source of bias in this study. Confounding occurs when the effect of an exposure of interest, in this case DL form, gets mixed with another variable that is associated with the exposure and the outcome [30], in this case healing times. Although the last Cochrane meta-analysis recommended pentavalent antimonial as the mainstay of cutaneous leishmaniasis treatment [31], due to the high failure rates of NMG in DL [5,10], clinical staff of our institution most frequently opted to use amphotericin in these cases. Limited data have shown a higher cure rate using liposomal amphotericin B in disseminated cases [10] compared to NMG in the same area [17]. Thus, it is possible that residual confounding associated with amphotericin use between groups is present in our results. This confounding, considering previous studies, seems more likely to have resulted in a reduced prognostic effect of DL form and not in the reversal of the direction of the association.

There is a lack of clinical trials comparing the use of pentavalent antimonials with amphotericin B in ATL, with international guidelines being formulated using observational studies [31,32]. In vitro, *L.* (*V*.) *braziliensis* seems to be sensitive to Amphotericin B [33,34]. There seems to be, however, great variation in the sensitivity of different isolates of this parasite to the drug [34,35,36]. This may account for the divergent cure rates observed in studies with this drug from different regions [36,37,38,39]. In a preliminary clinical trial from our region, a 50% cure rate was observed in patients treated with amphotericin B compared with 100% with N-methyl glucamine [38], which was similar to the 46% observed by Guery et al. in travelers who presented with new world CL [37]. Another important factor seems to be the low concentrations of Amphotericin reached in the skin by systemic administration, being significantly lower than in 11 of the 12 organs studied in a murine model [40]. Our results thus reinforce the importance of conducting large clinical trials to better understand the usefulness of this drug to treat ATL.

In clinical management, our findings in time-to-event analysis provide information that is directly relevant to patient care by estimating not only whether patients achieve cure but also how long healing is expected to take, which may facilitate patient counseling, improve follow-up planning, and help clinicians identify patients who may benefit from closer monitoring or earlier consideration of alternative treatment strategies. Future clinical trials should therefore consider incorporating time-to-event outcomes to improve the evaluation of therapeutic efficacy, particularly in uncommon clinical presentations such as disseminated leishmaniasis. Another strategy is the design of multicentric studies that may help to increase study power, better control for confounders and improve generalizability of results. The adoption of these important methodological improvements, however, is frequently not able to completely resolve the limitations related to small sample sizes of uncommon forms of the disease, as seen in our study.

## 5. Conclusions

In this retrospective cohort study, we analyzed the clinical outcomes of patients treated in our institution over a 10-year period using a time-to-event approach. Our findings suggest that DL is an important predictor of poorer prognosis, even after adjustment for age and sex. These results must, however, be interpreted with caution, especially in populations of different epidemiological backgrounds.

## Figures and Tables

**Table 1:** Characteristics of volunteers with CL and DL.

Characteristic	CL (n = 115)	DL (n = 8)	*p* Value
Sex (%)			
Female	41 (100%)	0 (0%)	0.05 *
Male	73 (90%)	8 (10%)	
Age (Years) Mean (sd)	44.6 (17.2)	45 (16.8)	0.94 †
			
Lesion duration (months)—Median (Q1–Q3; IQR)	4 (3–6.7–5.5; 3.75)	5 (4–5.5; 1.5)	0.71 ‡
			
Treatment (%)			
Amphotericin	12 (10.4%)	4 (50%)	0.01 *
Amphotericin	10 (8.7%)	2 (25%)	0.17 *
Glucantime	101 (87.8%)	7 (87.5%)	1 *
Pentamidine	4 (3.5%)	2 (25%)	0.04 *

(%): proportion; n: total of lesions; Median (Q1–Q3; IQR): Median and interquartile range (25th–75th percentiles). Mean (sd): Mean and standard deviation; * Fisher’s exact test, † Student’s *t*-test, ‡ Wilcoxon Rank Sum.

## Data Availability

The data presented in this study are available on request from the corresponding author.

## Abbreviations

The following abbreviations are used in this manuscript:

Abbreviation	Full Term
ATL	American tegumentary leishmaniasis
CL	Localized cutaneous leishmaniasis
DL	Disseminated Leishmaniasis
ML	Mucocutaneous leishmaniasis
DCL	Diffuse cutaneous leishmaniasis
HR	Hazard Ratios
IQR	Interquartile range
NMG	N-methyl glucamine
